# Distribution of Mother-to-Child Transmitted (MTCT) Infections and Socioeconomic Vulnerability Within the Gran Chaco Region

**DOI:** 10.3390/tropicalmed11070188

**Published:** 2026-07-08

**Authors:** Carla Rodríguez González, Susana Ávila, Karina Cardone, Mariana Fernández, Favio Crudo, Verónica Andreo, M. Victoria Periago

**Affiliations:** 1 The Instituto de Altos Estudios Espaciales Mario Gulich (CONAE-UNC), Cordoba X5186AAE, Argentina; veronica.andreo@ig.edu.ar; 2 National Scientific and Technical Research Council (CONICET), Buenos Aires C1033AAJ, Argentina; 3 Fundación Mundo Sano, Buenos Aires C1053ABK, Argentina

**Keywords:** mother-to-child transmission, Chagas disease, EMTCT plus, spatio-temporal analysis

## Abstract

Mother-to-child transmission (MTCT) of infectious diseases remains a public health challenge in socially vulnerable regions with limited healthcare access. This study assessed the epidemiological situation, spatial distribution, and socioeconomic context of MTCT infections—Chagas disease (ChD), syphilis, HIV, and hepatitis B (HB)—in the Gran Chaco region (Argentina–Paraguay), 2018–2024. Epidemiological data from 2877 patients enrolled in an MTCT Plus programme were analysed, alongside socioeconomic variables and spatio-temporal cluster analysis using SaTScan software. Maternal seroprevalence of ChD was 4.1%, the highest among the infections evaluated. Syphilis prevalence was 0.8%, while no HIV or HBV infections were detected among screened pregnant women. Two statistically significant spatiotemporal clusters of maternal *Trypanosoma cruzi* seropositivity were identified: a household-level cluster in 2018 and a regional cluster during 2019–2021. The highest prevalence of maternal ChD seropositivity was observed in census tracts with greater socioeconomic vulnerability, although this spatial overlap was assessed descriptively. These findings highlight the effectiveness of integrated maternal–child health services in ensuring coverage, timely diagnosis, and treatment in vulnerable populations. The identified spatial patterns provide evidence to support targeted surveillance and coordinated binational public health strategies in border regions affected by persistent social inequalities.

## 1. Author Summary

In this study, we investigated the distribution of infections with potential for MTCT—including Chagas disease, HIV, syphilis, and hepatitis B—in the Gran Chaco region of Argentina and Paraguay by combining epidemiological information, spatial analyses, and socioeconomic indicators.

Maternal seroprevalence of ChD was the highest among the infections evaluated, and two spatiotemporal clusters of maternal *T. cruzi* seropositivity were identified. Areas with higher maternal seroprevalence also exhibited greater socioeconomic vulnerability, although this relationship was assessed descriptively. Our findings demonstrate the value of integrated maternal and child healthcare programmes for achieving broad screening coverage, follow-up, and timely treatment, while providing evidence to guide surveillance and coordinated cross-border public health strategies in underserved populations.

## 2. Introduction

Mother-to-child transmission (MTCT) of HIV, syphilis, Hepatitis B virus (HBV), and Chagas disease (ChD) remains a significant public health concern in Latin America and the Caribbean (LAC), particularly in resource-limited settings where these diseases substantially contribute to morbidity and mortality [1,2]. According to the latest Pan American Health Organization’s (PAHO) reports for the region, approximately 2100 children per year are born with or acquire HIV from their mothers, 22,400 are infected with syphilis, 9000 are born with *T. cruzi*, the causative agent of ChD, and 6000 contract HBV infection [3]. Early detection of these infections during pregnancy enables effective therapeutic interventions for mothers and children. If left undiagnosed and untreated, they can lead to severe complications, including miscarriage, congenital and neurological disorders, cardiac conditions, and, in some cases, death [4].

Over the past two decades, the World Health Organization (WHO) and PAHO have led efforts to combat these diseases, initially focusing on HIV and syphilis and later expanding to include HB and ChD under the Framework for the Elimination of Mother-to-Child Transmission (EMTCT Plus) [5,6]. This initiative underscores the importance of a comprehensive approach to maternal and child health, ensuring timely access to diagnosis and treatment during pregnancy while promoting gender equity, empowerment, and the protection of human rights, including maternal health.

Despite the progress achieved through implementing the EMTCT Plus programme, reaching regional elimination targets remains challenging. Disparities in access to healthcare and other social determinants of health continue to hinder progress [7,8]. Although eight countries in the Americas have obtained WHO certification for the dual elimination of congenital HIV and syphilis, progress remains uneven. The regional MTCT rate of HIV stands at approximately 10%, far exceeding the target of 2%, while the incidence of congenital syphilis is 2 per 100,000 live births, surpassing the goal of 0.5. Moreover, ChD screening among pregnant women varies widely, with prevalence rates ranging from 7% to 55% [9,10].

The persistence of MTCT is closely linked to structural deficiencies in health systems, especially in the coverage and quality of prenatal care. Numerous studies have shown that delays in diagnosis, insufficient clinical follow-up, and inadequate treatment significantly increase the risk of vertical transmission [11,12]. Moreover, individual and social factors, such as maternal education level, employment status, ethnicity, and access to timely and adequate prenatal care, are known to compound this risk [13,14,15]. Generating context-sensitive evidence is key for guiding health authorities in designing and implementing more effective, equitable, and sustainable interventions.

The American Gran Chaco, particularly the Tri-Border region between Argentina, Bolivia, and Paraguay, is one of several areas where social and environmental determinants heavily influence health outcomes. It is marked by chronic poverty and a high proportion of indigenous and rural populations facing deep structural inequities. Factors such as food insecurity, poor access to clean water and sanitation, and precarious housing conditions all contribute to elevated maternal and infant morbidity and mortality [16,17].

Identifying geographical patterns and epidemiological trends is essential for guiding more effective and targeted public health strategies, enabling the optimisation of resources and the implementation of interventions directed at the most vulnerable populations [18,19]. Understanding the distribution of these infections not only strengthens the local health system but also facilitates evidence-based decision-making to advance the elimination of MTCT in contexts of high inequality and structural challenges. Here, we describe the spatial and temporal distribution of ChD, syphilis, HIV, and HBV infections in pregnant women in a region of the Gran Chaco, in relation to socioeconomic deprivation.

## 3. Methods

### 3.1. Study Area

**Figure 1:**
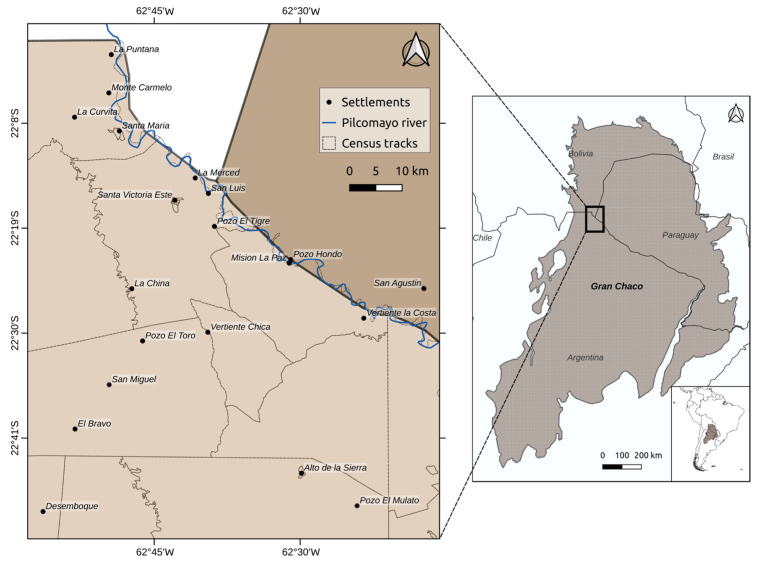
Study area within the Gran Chaco Region in the tri-border between Argentina, Bolivia, and Paraguay (right quadrant). Map detailing the settlements in the border regions between the three countries (left quadrant). Maps created with QGIS 3.4 open-source software.

The study area encompassed a transboundary Gran Chaco region, including Santa Victoria Este and Alto la Sierra in Argentina, D’Orbigny in Bolivia, and Pozo Hondo and San Agustín in Paraguay (Figure 1). This region, with an approximate area of 7600 km^2^ and an estimated population of 13,500 inhabitants according to the most recent census data [20], is characterised as a cultural mosaic of indigenous communities that have historically converged there due to migratory processes. The population distribution is highly heterogeneous and is largely determined by water availability and frequent overflows of the Pilcomayo River that pose recurrent challenges to local communities.

The region has a subtropical climate, with a dry season from April to December and a rainy season during the remaining months. Annual precipitation varies between 500 and 700 mm. Temperatures vary widely, often exceeding 40 °C in summer, while in the south and southwest, winter temperatures can drop below 0 °C [16]. The combination of flooding and prolonged droughts further exacerbates environmental and socio-economic vulnerabilities. Additionally, the predominant xerophytic forest vegetation has been largely replaced by agriculture and livestock farming, resulting in landscape transformation and competition for resources [16].

### 3.2. Data

#### 3.2.1. Epidemiological Data

The epidemiological data were collected between 2018 and 2024 in the framework of the EMTCT Plus initiative, implemented by two non-governmental organizations (NGOs), Fundación Mundo Sano (FMS) and Asociación para el Desarrollo Sanitario Regional (ADESAR), in collaboration with local, regional, and national health authorities. This initiative has been implemented since 2018 to support the elimination of MTCT of HIV, syphilis, HB, and ChD in vulnerable populations across the Tri-Border region [21,22,23]. A specialised team provided antenatal care during periodic field visits conducted approximately every 60 days. Each visit consisted of an intensive five-day intervention period, during which the team performed antenatal care activities, supported by local healthcare professionals and health system actors who ensured continuity of care between visits. Data collection was conducted at health posts in seven localities within the municipality of Santa Victoria Este and the locality of Alto la Sierra, Rivadavia Department, Salta Province (Argentina), as well as at two health posts in the localities of Pozo Hondo and San Agustín, Boquerón Department (Paraguay). Demographic information, including age, sex, ethnicity, and household distribution, was recorded for all pregnant, postpartum, and breastfeeding women. Additionally, local medical records were collected to document the follow-up and management of HIV, syphilis, HBV, and ChD in the aforementioned groups, as well as in children, siblings, and/or partners of pregnant women enrolled in the implementation.

The diagnosis and clinical management of HIV, syphilis, HBV, and *T. cruzi* infection followed the national diagnostic algorithms recommended by the Argentine Ministry of Health [24]. Maternal HIV, syphilis, and HBV infections were diagnosed using the recommended serological algorithms, including HBsAg detection for HBV. Maternal *T. cruzi* infection was confirmed by conventional serology using two assays based on different analytical principles. Congenital ChD was diagnosed according to the national algorithm using parasitological methods and/or real-time PCR during the neonatal period, or by conventional serology after 10 months of age when early tests were negative.

Details on the study setting and population demographics have been published elsewhere [22].

#### 3.2.2. Case Distribution

Confirmed cases were georeferenced at the household level using a GPS device (Garmin eTrex 32x; Garmin Ltd., Olathe, KS, USA). Geographic coordinates were collected using the Universal Transverse Mercator (UTM) projection, zone 21S. These operations covered over 15 settlements within the study area and involved collaboration with nursing staff, community health agents, and local community members.

#### 3.2.3. Socioeconomic Data

Socioeconomic data were obtained from the most recent population census in Argentina and Paraguay (2022) [20,25] and correspond to the Unmet Basic Needs (UBN) index, which identifies households experiencing deprivation in at least one of the following categories: housing conditions (precarious or non-residential dwellings), sanitary conditions (lack of a toilet), overcrowding (more than three persons per room), school attendance (at least one child aged 6–12 not attending school), and subsistence capacity (high dependency ratio and low educational attainment of the household head). In addition to these categories, we also considered specific variables related to housing conditions, including households without access to piped water for drinking and cooking, households without a type 1 roof covering (membrane, tile, slab, or shingle), and households without a type 1 floor covering (ceramic, tile, mosaic, marble, wood, or carpet).

### 3.3. Data Processing

#### 3.3.1. Socioeconomic Analysis

To explore the spatial relationship between infection distribution and socioeconomic conditions, thematic maps were generated at the census tract level for the UBN index and its individual components. The spatial distribution of maternal *T. cruzi* seropositivity and syphilis cases was then descriptively compared with these socioeconomic indicators to identify areas of potential spatial co-occurrence between infection and social deprivation. No formal statistical analyses were performed; therefore, this component of the study was intended as a descriptive spatial assessment rather than an inferential analysis of association.

#### 3.3.2. Spatio-Temporal Clustering Patterns

Clustering patterns of cases for the four infections studied were analysed using a discrete Poisson space-time scan model implemented in the SaTScan software v10.3 (Information Management Services, Inc., Rockville, MD, USA) [26]. The analysis included 115 confirmed positive cases distributed across the study area, with the total population of each settlement as the at-risk population. Geographical coordinates from 74 households were used to locate cases within the study area. It was assumed that the number of cases in each location follows a Poisson distribution, where the expected number of cases in an area is proportional to its population size. This approach allows for the detection of statistically significant clusters by comparing observed case counts with expected values under the null hypothesis of random spatial and temporal distribution. The analysis incorporated a space-time scan statistic, which enables the identification of clusters that vary both spatially and temporally, providing insights into potential outbreak patterns and temporal trends. Additionally, the locations of the detected clusters were compared with the UBN index to assess correlations between high disease incidence and socioeconomic deprivation.

## 4. Results

### 4.1. Epidemiological Situation

This study included 2877 patients, comprising pregnant women, postpartum women, newborns, and other children. Between 2018 and 2024, the private–public healthcare implementation provided a substantial number of services, including 2575 controlled pregnancies and approximately 3900 prenatal check-ups.

Regarding infections within the EMTCT Plus framework, the evaluation of 2575 pregnant women revealed that ChD was the most prevalent condition, with 4.1% of the women testing positive. ChD cases were also detected in newborns and preexisting children born to mothers with the disease. In contrast, syphilis was only identified among pregnant women, with a prevalence of 0.8%. Notably, no cases of HIV or HBV were reported during the study period.

Among preexisting children of mothers with ChD, 205 out of 236 (86.9%) were tested, with 14 (6.8%) diagnosed as positive for *T. cruzi* infection. Of the affected children, 12 (85.7%) received treatment. In the case of newborns, 97 out of 111 (87.4%) were tested, and 11 (11.3%) were diagnosed with *T. cruzi* infection. All infected newborns (100%) received treatment.

### 4.2. Distribution of Chagas and Syphilis Cases

**Figure 2:**
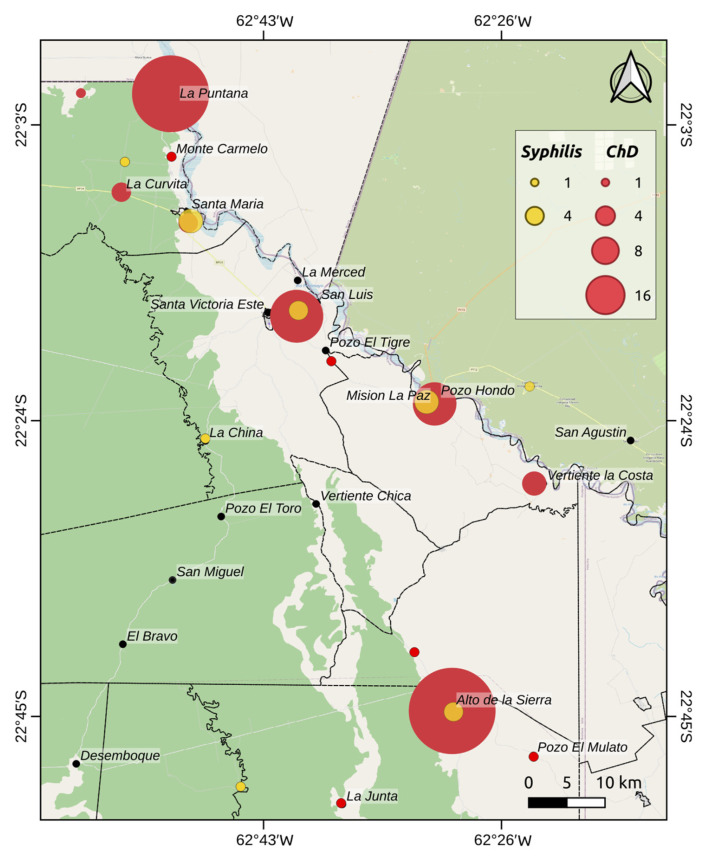
Distribution of Chagas Disease (in red) and Syphilis (in yellow) cases by settlement in the border area between Argentina and Paraguay, Gran Chaco Region. The diameter of each circle represents the number of households with positive cases aggregated by settlement.

The distribution and prevalence of *T. cruzi* and syphilis among tested women between 2018 and 2024 varied according to settlement and country (Table 1). In Paraguay, data were available only for Pozo Hondo, where *T. cruzi* was detected in 10.0% of the tested women, while syphilis was found in 15.0%. In Argentina, the settlements with the highest prevalence of *T. cruzi* were La Junta (100.0%), San Luis (14.8%), Vertiente de la Costa (14.1%), and La Puntana (11.6%). In Alto la Sierra and Misión La Paz, infection rates were 11.3% and 6.6%, respectively. In contrast, Santa María and Pozo El Tigre exhibited the lowest rates, with 2.0% and 3.4%, respectively. Syphilis was detected in several settlements, with prevalence ranging from 1.3% (El Cañaveral) to 6.3% (Pozo La China). In Santa María and Alto la Sierra, rates of 2.5% and 2.1% were recorded, respectively. The “Others” category in Table 1 includes women tested in several smaller settlements throughout the study area whose residences were not assigned to a specific settlement at the time of data collection. Therefore, these records were grouped into a single category for descriptive purposes. Figure 2 illustrates the spatial distribution of both diseases in the study area.

### 4.3. Socioeconomic Analysis

**Figure 3:**
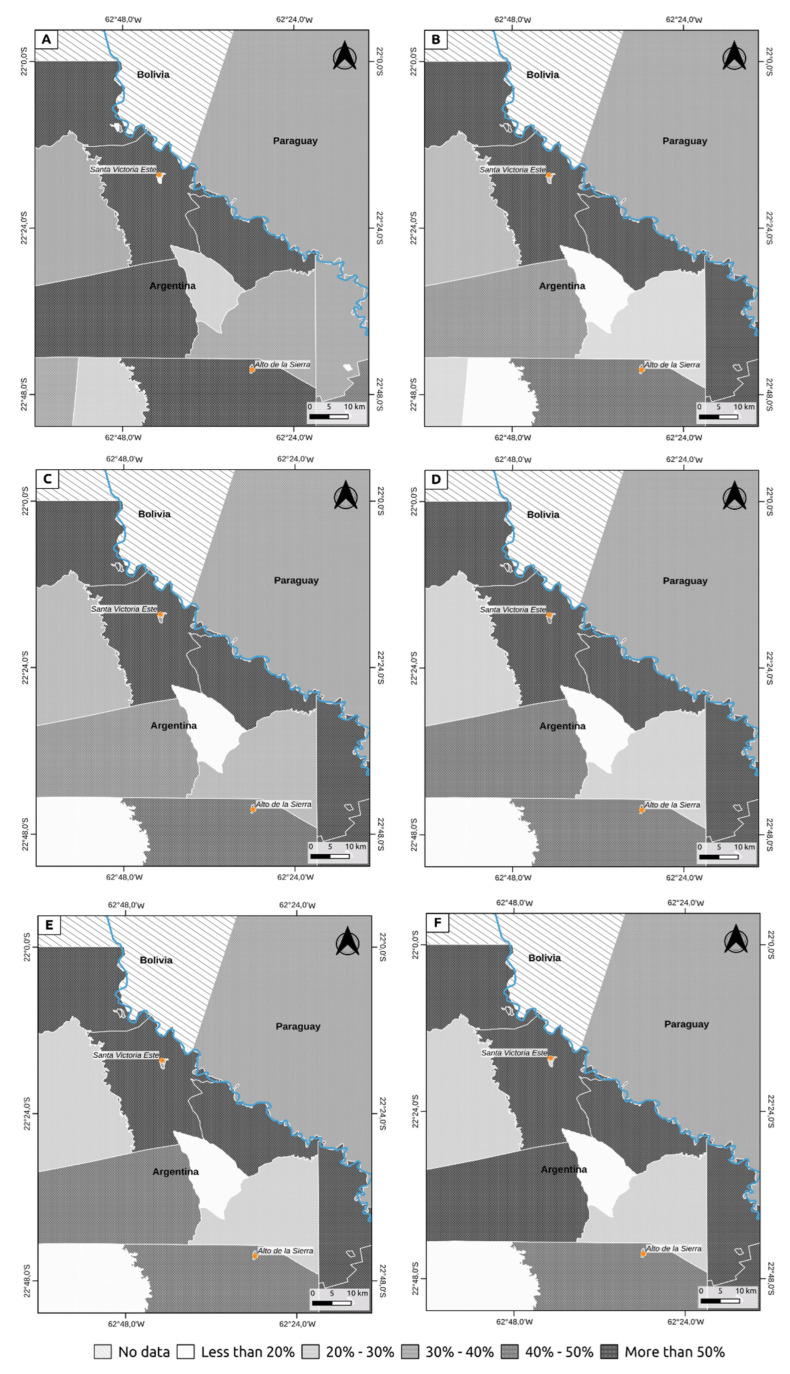
Socioeconomic situation in the Gran Chaco Region between Argentina and Paraguay. (**A**) Households without access to piped water for drinking and cooking; (**B**) Households experiencing critical overcrowding (more than three persons per room); (**C**) Households without a flushing toilet system (button, chain, or tank); (**D**) Households without a type 1 roof covering (membrane, tile, slab, or shingle); (**E**) Households without a type 1 floor covering (ceramic, tile, mosaic, marble, wood, or carpet); (**F**) Households classified as having Unmet Basic Needs (UBN).

The analysis of various socioeconomic variables at the census tract level indicated that the highest levels of social vulnerability were observed in the Argentine communities of the Tri-Border region (Figure 3). The census tracts along the banks of the Pilcomayo River accounted for more than 80% of the total analysed population. In these areas, more than 50% of the population was found to have at least one unmet basic need, such as access to domestic piped water, severe overcrowding, and substandard housing conditions. The census tract of Alto la Sierra was also found to exhibit high levels of vulnerability, particularly concerning access to piped water and the availability of a flushing toilet system.

### 4.4. Spatio-Temporal Clustering Patterns

**Figure 4:**
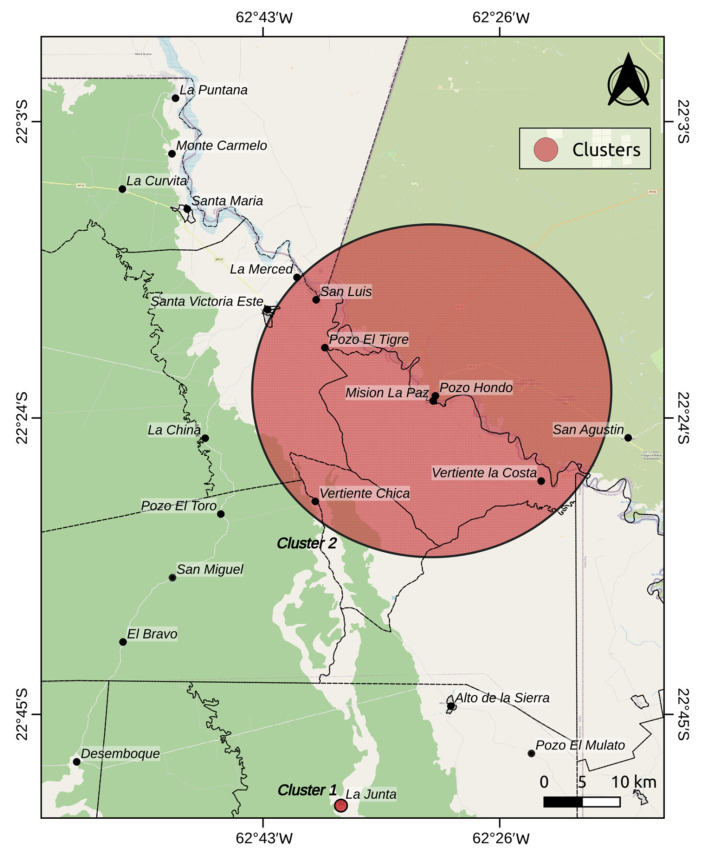
Statistically significant spatio-temporal clusters of ChD cases in the Gran Chaco Region between Argentina and Paraguay.

Non-random patterns were identified in the distribution of ChD cases, indicating spatio-temporal clustering. Two statistically significant, non-overlapping clusters were identified (*p*-value < 0.05, Table 2). Cluster 1, with a radius of 0 km, grouped four cases within a single household located in La Junta during the second half of 2018. This cluster exhibited the highest relative risk (RR = 132.43), far exceeding the expected number of cases (0.031), and showed a highly significant *p*-value (*p* < 0.001). Cluster 2, identified between 2019 and 2021, encompassed 33 cases within a radius of 21.02 km, distributed across five settlements in Argentina—Vertiente de la Costa, Misión La Paz, Vertiente Chica, Pozo el Tigre, and San Luis—as well as one settlement in Paraguay, Pozo Hondo (Figure 4). Although its relative risk was lower (RR = 2.71), it was still statistically significant (*p* = 0.01) and represented a substantial excess over the expected number of cases (14.89). No significant spatial clusters were found for syphilis cases.

## 5. Discussion

The findings of this study provide updated evidence on the distribution and dynamics of MTCT infections in a region characterised by high structural vulnerability and unequal access to healthcare services. In this context, a key finding was the identification of a substantial maternal seroprevalence of ChD (4.1%), together with the detection of two spatiotemporal clusters of cases: a household-level cluster in 2018 and a regional cluster between 2019 and 2021. These patterns suggest areas where *T. cruzi* infection remains concentrated and highlight the need for targeted binational strategies, particularly in areas characterised by high socioeconomic vulnerability.

A broad coverage of maternal and child healthcare services was achieved throughout the study period, as evidenced by the volume of prenatal care provided and the number of patients tested and treated. A total of 2575 pregnancies were monitored, and approximately 3900 prenatal consultations were conducted, indicating that access to essential services was successfully ensured for a substantial portion of the target population (n = 2877).

Within the framework of the EMTCT Plus initiative, screening for congenital infections among pregnant women revealed that ChD was the most prevalent condition, with a positivity rate of 4.1%. This finding is consistent with the endemic nature of *T. cruzi* infection in the region and highlights the ongoing risk of vertical transmission [27,28,29]. Similar studies in other endemic regions have reported comparable maternal seroprevalence rates and associated adverse neonatal outcomes, reinforcing the urgency of including *T. cruzi* in mandatory pregnancy screening programs [30,31]. Among children born to mothers with ChD, high testing coverage was achieved, and all confirmed congenital cases received timely treatment (100% treatment rate), illustrating the effectiveness of integrated maternal and child healthcare pathways. Nevertheless, cases confirmed only by serology after 10 months of age should be interpreted with caution, as congenital and early postnatal vector-borne transmission cannot be fully distinguished with the available data.

In contrast, syphilis was detected at a substantially lower rate (0.8%), and no cases of HIV or HBV were identified despite the approximately 95% screening coverage achieved among pregnant women. For HBV, this finding is consistent with the long-standing implementation of universal infant hepatitis B vaccination and routine antenatal screening in Argentina, which have substantially reduced the burden of HBV infection among women of reproductive age. For HIV, the absence of detected cases may reflect a genuinely low prevalence in the study population; however, the sample size limits the ability to detect rare events, and undiagnosed infections, including those occurring during the serological window period, cannot be completely excluded. Therefore, these findings should be interpreted as consistent with a low prevalence of HIV and HBV in the study population rather than as definitive evidence of their absence.

Distinct spatial patterns of ChD and syphilis prevalence were identified across settlements, requiring contextual interpretation in light of their differing epidemiological characteristics and associated sociodemographic factors [32,33,34]. As shown in Table 1, the highest prevalence of maternal ChD was recorded in census tracts located along the Pilcomayo River and near Alto la Sierra—areas characterised by higher population density. This spatial concentration of maternal ChD cases may reflect the historical endemicity of *T. cruzi* infection in these areas, together with persistent structural vulnerabilities and limited access to timely diagnosis and treatment [35,36]. Regarding the spatial distribution of syphilis, cases were more evenly distributed across the study area. Although the highest settlement-specific prevalence was observed in Pozo Hondo (15.0%; 3/20 women tested), this estimate should be interpreted with caution because it was based on a very small number of women and no statistically significant spatial cluster was identified in the SaTScan analysis. Consequently, this finding is more likely to reflect the small sample size than a true local hotspot, although additional surveillance in Pozo Hondo would help determine whether a higher prevalence is present. The detection of syphilis cases in multiple settlements, despite the low overall prevalence, nevertheless highlights the importance of maintaining comprehensive antenatal screening and strengthening sexual and reproductive health services throughout the region.

A descriptive comparison between the spatial distribution of positive cases and socioeconomic indicators suggested a pattern of co-occurrence with social deprivation. As illustrated in Figure 3, the census tracts located along the Pilcomayo River, where the highest prevalence of maternal *T. cruzi* seropositivity and syphilis was recorded, also exhibited the highest levels of unmet basic needs. Critical overcrowding (Figure 3B), lack of flushing toilet systems (Figure 3C), and poor housing conditions, including substandard roofing and flooring materials (Figure 3D,E), were consistently observed in these areas. Previous studies have associated these socioeconomic conditions with an increased risk of vector-borne and sexually transmitted infections through greater environmental exposure and reduced access to hygiene and healthcare services [35,37,38,39]. Likewise, Alto la Sierra presented multiple housing-related vulnerabilities. Although the spatial overlap between positive cases and structural deprivation, summarized by the Unmet Basic Needs Index (Figure 3F), is consistent with the broader literature on the social determinants of health, these findings should be interpreted as descriptive observations rather than evidence of a statistical association. Further studies incorporating formal statistical analyses and entomological data are needed to better understand the relationship between socioeconomic vulnerability and the spatial distribution of these infections.

The spatiotemporal analysis provided additional insight into the spatial and temporal distribution of maternal *T. cruzi* seropositivity by identifying two statistically significant clusters, each with distinct characteristics in terms of magnitude, geographic extent, and temporal distribution (Table 2, Figure 4). Cluster 1, concentrated within a single household in La Junta, exhibited an exceptionally high relative risk and may reflect a localized aggregation of cases in a setting of extreme social and environmental vulnerability. Although the detection of multiple cases within one household is compatible with possible intradomiciliary transmission, the absence of entomological data precludes confirmation of the underlying transmission mechanism. Cluster 2, spanning multiple settlements across Argentina and Paraguay, represented a broader cross-border spatial pattern. Although its relative risk was lower than that of Cluster 1, the number of observed cases substantially exceeded the expected number, indicating a persistent spatial concentration of maternal *T. cruzi* seropositivity during 2019–2021.

Previous studies have shown that the spatial distribution of *T. cruzi* infection is influenced by ecological factors. In northern Argentina and Chile, fine-scale analyses demonstrated that infestation and infection patterns were more closely associated with land cover and host dynamics than with administrative boundaries, highlighting the importance of geographically targeted interventions in environmentally and socially vulnerable border regions [40,41]. By contrast, no statistically significant clusters of syphilis cases were identified. This finding may reflect both the distinct epidemiology of syphilis, which is strongly influenced by human mobility and sexual networks [34,37], and the limited number and geographic dispersion of cases detected in the present study (n = 23), which reduced the statistical power to detect localized clusters [42,43]. Therefore, the absence of significant clusters should not be interpreted as evidence that localized clusters of syphilis cases do not exist, but rather that the available data were insufficient to identify them. Longer surveillance periods and larger datasets will be needed to better characterize the spatial distribution of syphilis in this region.

Despite these contributions, important challenges remain for research and surveillance in this region. The absence of data from the Bolivian side of the tri-border area reflects the broader difficulties of data sharing and surveillance integration across international borders, limiting the ability to fully characterize transmission dynamics in this highly connected epidemiological setting. In addition, although the screening strategy achieved approximately 95% coverage of pregnant women in the participating communities, the NGO-led recruitment approach may have introduced some degree of selection bias, as women who were not reached or who did not participate may have differed from those included in the study. Nevertheless, the high screening coverage likely reduced the magnitude of this potential bias. Strengthening cross-border surveillance systems and harmonizing data collection between neighboring countries will be essential to improve the identification of priority areas for intervention and to support coordinated prevention, follow-up, and treatment strategies.

Building on the findings of the present study, future research should aim to directly estimate congenital transmission rates and identify the maternal, parasitological, environmental, and socioeconomic factors associated with transmission. Incorporating systematic molecular diagnosis in newborns, extending follow-up periods, and applying formal spatial and statistical analyses would complement the descriptive and spatiotemporal approaches presented here, providing a more comprehensive understanding of the determinants and geographic distribution of MTCT infections in this vulnerable border region.

## 6. Conclusions

The findings of this study highlight the effectiveness of an integrated maternal and child healthcare strategy in a context of high social vulnerability, as demonstrated by the broad screening coverage, follow-up, and timely treatment achieved within the EMTCT Plus initiative. The identification of distinct spatial patterns of maternal *T. cruzi* seropositivity and their co-occurrence with areas of greater structural deprivation underscores the importance of sustaining targeted interventions that address both healthcare access and the broader social determinants of health. In addition, the detection of spatiotemporal clusters identified areas where *T. cruzi* infection remained concentrated over time, supporting the need for coordinated binational surveillance and control strategies in border regions facing shared epidemiological challenges. Strengthening surveillance systems, ensuring equitable access to early diagnosis and treatment, and promoting cross-border collaboration will be essential to advance the elimination of mother-to-child transmission of these infections. Overall, this study provides valuable evidence to support the development of more equitable, context-sensitive public health policies aimed at reducing health disparities in historically underserved populations.

## Figures and Tables

**Table 1:** Prevalence of *T. cruzi* and Syphilis cases among tested women by settlement in Paraguay and Argentina (2018–2024). Values are presented as *n* (%); 95% confidence intervals (Wilson score) are shown in parentheses.

Country	Settlement	Total Tested Women	Number of *T. cruzi* Positive (%) [95% CI]	Number of Syphilis Positive (%) [95% CI]
Paraguay	Pozo Hondo	20	2 (10.0, 2.8–30.1)	3 (15.0, 5.2–36.0)
	San Agustin	71	-	-
Argentina	La Merced	44	-	2 (4.5, 1.3–15.2)
	Misión la Paz	226	15 (6.6, 4.1–10.5)	4 (1.8, 0.7–4.5)
	Vertiente de la Costa	71	10 (14.1, 7.8–24.1)	-
	Pozo La China	16	-	1 (6.3, 1.1–28.3)
	San Luis	54	8 (14.8, 7.7–26.6)	-
	Santa María	203	4 (2.0, 0.8–4.9)	5 (2.5, 1.1–5.6)
	Pozo El Tigre	59	2 (3.4, 0.9–11.5)	1 (1.7, 0.3–9.0)
	Pozo El Toro	21	-	-
	La Puntana	241	28 (11.6, 8.1–16.2)	-
	Monte Carmelo	31	1 (3.2, 0.6–16.2)	1 (3.2, 0.6–16.2)
	La Curvita	76	4 (5.3, 2.1–12.8)	-
	Santa Victoria Este/El Cañaveral	79	9 (11.4, 6.1–20.4)	1 (1.3, 0.2–6.8)
	Vertiente Chica	1	-	-
	Alto la Sierra	239	27 (11.3, 7.8–16.1)	5 (2.1, 0.9–4.8)
	Desemboque	5	-	-
	El Bravo	9	-	-
	La Junta	4	4 (100, 51.0–100.0)	-
	Pozo el Mulato	16	1 (6.3, 1.1–28.3)	-
	Others *	1087	-	-

* Others: Includes women tested in smaller settlements whose residences were not assigned to a specific settlement at the time of data collection. This category comprises Sauzalito, San Bernardo, Bajo Grande, Pozo El Mulato, La Esperanza, Pozo El Bravo, Las Mojarras, La Carneada, Morón, and Pozo Los Ranchos.

**Table 2:** Clusters detected for the distribution of Chagas disease cases using the discrete Poisson model.

Clusters Detected	C luster 1	Cluster 2
Overlap with clusters	No Overlap	No Overlap
Coordinates/radius	(22.85 S, 62.62 W)/0 km	(22.36 S, 62.52 W)/21.02 km
Time frame	1 June 2018 to 31 December 2018	1 January 2019 to 31 December 2021
Number of cases	4	33
Expected cases	0.031	14.89
Annual cases/100,000	114,631.9	1986.6
Observed/expected	127.86	2.22
Relative risk	132.43	2.71
Log likelihood ratio	15.50	9.89
*p*-value	0.000062 ***	0.01 *

Note: * *p* ≤ 0.01; *** *p* ≤ 0.001.

## Data Availability

The datasets used and/or analysed during the current study are available from the corresponding author on reasonable request.
